# Temporal Identification of Dysregulated Genes and Pathways in Clear Cell Renal Cell Carcinoma Based on Systematic Tracking of Disrupted Modules

**DOI:** 10.1155/2015/313740

**Published:** 2015-10-12

**Authors:** Shao-Mei Wang, Ze-Qiang Sun, Hong-Yun Li, Jin Wang, Qing-Yong Liu

**Affiliations:** ^1^Center for Kidney Disease, Jinan Central Hospital Affiliated to Shandong University, Jinan, Shandong 250013, China; ^2^Department of Urinary Surgery, Qianfoshan Hospital Affiliated to Shandong University, 16766 Jingshi Road, Jinan, Shandong 250014, China

## Abstract

*Objective*. The objective of this work is to identify dysregulated genes and pathways of ccRCC temporally according to systematic tracking of the dysregulated modules of reweighted Protein-Protein Interaction (PPI) networks. *Methods*. Firstly, normal and ccRCC PPI network were inferred and reweighted based on Pearson correlation coefficient (PCC). Then, we identified altered modules using maximum weight bipartite matching and ranked them in nonincreasing order. Finally, gene compositions of altered modules were analyzed, and pathways enrichment analyses of genes in altered modules were carried out based on Expression Analysis Systematic Explored (EASE) test. *Results*. We obtained 136, 576, 693, and 531 disrupted modules of ccRCC stages I, II, III, and IV, respectively. Gene composition analyses of altered modules revealed that there were 56 common genes (such as *MAPK1*, *CCNA2*, and *GSTM3*) existing in the four stages. Besides pathway enrichment analysis identified 5 common pathways (glutathione metabolism, cell cycle, alanine, aspartate, and glutamate metabolism, arginine and proline metabolism, and metabolism of xenobiotics by cytochrome P450) across stages I, II, III, and IV. *Conclusions*. We successfully identified dysregulated genes and pathways of ccRCC in different stages, and these might be potential biological markers and processes for treatment and etiology mechanism in ccRCC.

## 1. Introduction

Clear cell renal cell carcinoma (ccRCC) is the most common type of kidney cancer and accounts for approximately 60% to 70% of all renal tumors [1]. Patients with ccRCC comprise a heterogeneous group of patients with variable pathologic stage and grade, used to stratify patients and infer prognosis [2]. However, providing patients with reliable information about anticipated treatment response is challenging due to the molecular heterogeneity of ccRCC [3]. Delineating the pathogenesis of ccRCC by investigating the gene and epigenetic changes and their effects on key molecules and their respective biologic pathways is of crucial importance for the improvement of current diagnostics, prognostics, and drug development [4]. For example, studies suggest that ccRCC is closely associated with tumor suppressor von-Hippel Lindau (*VHL*) gene mutations that lead to stabilization of hypoxia inducible factors (HIF-1*α* and HIF-2*α*, also known as HIF1A and EPAS1) in both sporadic and familial forms [5, 6].

With the advances of high-throughput experimental technologies, large amounts of Protein-Protein Interaction (PPI) data are uncovered, which make it possible to study proteins on a systematic level [7, 8]. In addition, a PPI network can be modeled as an undirected graph, where vertices represent proteins and edges represent interactions between proteins, to prioritize disease associated genes or pathways and to understand the modus operandi of disease mechanisms [9, 10]. But it has been noticed that PPI data are often associated with high false positive and false negative rates due to the limitations of the associated experimental techniques and the dynamic nature of protein interaction maps, which may have a negative impact on the performance of complex discovery algorithms [11]. Many computational approaches have been proposed to assess the reliability of protein interactions data. An iterative scoring method proposed by Liu et al. [12] was selected to evaluate the reliability and predict new interactions, and it has been shown to perform better than other methods. However, studying multiple diseases simultaneously makes it challenging to discern clearly the intricate underlying mechanisms.

In addition, it is important to effectively integrate omics data into such an analysis; for example, Chu and Chen [13] combined PPI and gene expression data to construct a cancer perturbed PPI network in cervical carcinoma to study gain- and loss-of-function genes as potential drug targets. Magger et al. [14] combined PPI and gene expression data to construct tissue-specific PPI networks for 60 tissues and used them to prioritize disease genes. Beyond straightforward scoring genes in the gene regulatory network, it is crucial to study the behavior of modules across specific conditions in a controlled manner to understand the modus operandi of disease mechanisms and to implicate novel genes [15], since some of the important genes may not be identifiable through their own behavior, but their changes are quantifiable when considered in conjunction with other genes (e.g., as modules). What is required, therefore, is systematic tracking gene, pathways, and module behavior across specific conditions in a controlled manner.

Therefore, in this paper, we performed a temporal (stages I, II, III, and IV of ccRCC) analysis between normal controls and ccRCC patients to identify disrupted genes and pathways by systematically tracking the altered modules of reweighted PPI network. To achieve this, we firstly inferred normal and ccRCC cases of different stages PPI networks based on Pearson correlation coefficient (PCC); next, clique-merging algorithm was performed to explore modules in PPI network, and we compared these modules to identify altered modules; then gene composition of these modules was analyzed; finally, pathways enrichment analysis of genes in altered modules was carried out based on Expression Analysis Systematic Explored (EASE) test.

## 2. Materials and Methods

### 2.1. Inferring Normal and ccRCC PPI Network

#### 2.1.1. PPI Network Construction

We utilized a dataset of human PPI network, the Search Tool for the Retrieval of Interacting Genes/Proteins (STRING), which comprised 16730 genes and 1048576 interactions [16]. For STRING, self-loops and proteins without expression value were removed. The remaining largest connected component with score of more than 0.8 was kept as the selected PPI network, which consisted of 8590 genes and 53975 interactions.

#### 2.1.2. Gene Expression Dataset and Dataset Preprocessing

A microarray expression profile, E-GEOD-53757, from Array Express database was selected for ccRCC related analysis. E-GEOD-53757 which existed on Affymetrix GeneChip Human Genome U133 Plus 2.0 Platform was divided into 4 groups according to tumor stage (stages I, II, III, and IV). There were 24, 19, 14, and 15 ccRCC patients at stages I, II, III, and IV, respectively; the number of normal controls in each stage was equaled to its patients' number.

The expression profile was preprocessed by standard methods, consisting of “rma” [17], “quantiles” [18], “mas” [19], and “medianpolish” [17]. To be specific, “rma” method was carried out for background correction to eliminate influences of nonspecific hybridization [17]. The quantile normalization algorithm was a specific case of the transformation *x*_*i*_′ = *F* ^−1^(*G*(*x*_*i*_)), where we estimated *G* by the empirical distribution of each array and *F* using the empirical distribution of the averaged sample quantiles [18]. Perfect match (PM)/mismatch (MM) correction was conducted by “mas” method [19]. Summarization of the probe data was conducted by “medianpolish” [17]. A multichip linear model was fit to data from each probe set. In particular, for a probe set *k* with *i* = 1,…, *I*_*k*_ probes and data from *j* = 1,…, *J* arrays, we fitted the following model, log_2_(PM_*ij*_^*k*^) = *α*_*i*_^*k*^ + *β*_*j*_^*k*^ + *ε*_*ij*_^*k*^, where *α*_*i*_ was a probe effect and *β*_*j*_ was the log_2_ expression value.

Next, the data were screened by feature filter method of gene filter package, and the number of genes with multiple probes was 20102. At last, we obtained the gene expression value for each gene, including 20102 genes from 144 samples (72 normal controls and 72 ccRCC patients).

#### 2.1.3. Reweighting Gene Interactions by PCC

Gene interactions in network based on ccRCC patients of different stages (stages I, II, III, and IV) and their normal controls were reweighted by PCC, which evaluated the probability of two coexpressed gene pairs. PCC is a measure of the correlation between two variables, giving a value between −1 and +1 inclusively [20]. The PCC of a pair of genes (*x* and *y*), which encoded the corresponding paired proteins (*u* and *v*) interacting in the PPI network, was defined as

(1) PCCx,y=1s−1∑i=1sgx,i−g¯xσx·gy,i−g¯yσy,

where *s* was the number of samples of the gene expression data; *g*(*x*, *i*) or *g*(*y*, *i*) was the expression level of gene *x* or *y* in the sample *i* under a specific condition; $\bar{g}(x)$ or $\bar{g}(y)$ represented the mean expression level of gene *x* or *y*; and *g*(*x*) or *g*(*y*) represented the standard deviation of expression level of gene *x* (or *y*).

The PCC of a pair of proteins (*u* and *v*) was defined as the same as the PCC of their corresponding paired genes (*x* and *y*), which was PCC(*u*, *v*) = PCC(*x*, *y*). If PCC(*u*, *v*) has a positive value, there is a positive linear correlation between *u* and *v*. In addition, we defined PCC of each gene-gene interaction as weight value of the interaction.

### 2.2. Identifying Modules from the PPI Network

In this paper, module-identification algorithm is based on clique-merging [21, 22] and is similar to the method proposed by Liu et al. [12]. It consisted of three steps; in the first step, it found all the maximal cliques from the weighted PPI network. Maximal cliques were evaluated by a fast depth-first search with pruning-based algorithm proposed by Tomita et al. [23]. It utilized a depth-first search strategy to enumerate all maximal cliques and effectively pruned nonmaximal cliques during the enumeration process.

In the second step, we assigned a score to each clique; the score of a clique *C* was defined as its weighted density *d*_*W*_(*C*):

(2) $$\begin{array}{c} d_{W}\left(\;C\;\right)=\frac{\sum_{u\in C,v\in C}w\left(u,v\right)}{\left|C\right|\cdot \left(\left|C\right|-1\right)}, \end{array}$$

where *w*(*u*, *v*) was the weight of the interaction between *u* and *v*. We ranked these cliques in nonincreasing order of their weighted densities {*C*_1_, *C*_2_,…, *C*_*k*_}.

Finally, we went through this ordered list repeatedly merging highly overlapping cliques to build modules. For every clique *C*_*i*_, we repeatedly looked for a clique *C*_*j*_ (*j* > *i*) such that the overlap |*C*_*i*_∩*C*_*j*_ | /|*C*_*j*_ | ≥*t*, where *t* = 0.5 was a predefined threshold for overlapping [15]. If such *C*_*j*_ was found, we calculated the weighted interconnectivity *I*_*w*_ between *C*_*i*_ and *C*_*j*_ as follows:

(3) IwCi,Cj=∑u∈Ci−Cj∑v∈Cjwu,vCi−Cj·Cj·∑u∈Cj−Ci∑v∈Ciwu,vCj−Ci·Ci.

If *I*_*w*_(*C*_*i*_, *C*_*j*_) ≥ *t*, then *C*_*j*_ was merged into *C*_*i*_ forming a module; else *C*_*j*_ was discarded.

We captured the effect of differences in interaction weights between normal and ccRCC cases through the weighted density-based ranking of cliques. Weighted density assigned higher rank to larger and stronger cliques. Therefore, we expected cliques with lost proteins or weakened interactions to go down the rankings resulting in altered module generation, thereby capturing changes in modules between normal and ccRCC cases.

### 2.3. Comparing Modules between Normal and ccRCC Conditions

The approach to compare modules between normal and ccRCC conditions is similar to the method proposed by Srihari and Ragan [15]. In detail, *H*_*N*_ and *H*_*T*_ represented the PPI network of normal controls and ccRCC patients, identifying the sets of modules *S* = {*S*_1_, *S*_2_,…, *S*_*m*_} and *T* = {*T*_1_, *T*_2_,…, *T*_*n*_}, respectively. For each *S*_*i*_ ∈ *S*, module correlation density *d*_*c*_(*S*_*i*_) was defined as

(4) $$\begin{array}{c} d_{c}\left(\;S_{i}\;\right)=\frac{\sum_{x,y\in S_{i}}\text{PCC}\left(\left(x,y\right),M\right)}{\left|S_{i}\right|\cdot \left(\left|S_{i}\right|-1\right)}. \end{array}$$

Correlation densities of ccRCC modules (*d*_*c*_(*T*_*i*_)) were calculated similarly.

Disrupted or altered module pairs were evaluated by modeling the set *ϒ*(*S*, *T*) as maximum weight bipartite matching [24]. Firstly, we build a similarity graph *M* = (*V*_*M*_, *E*_*M*_), where *V*_*M*_ = {*S* ∪ *T*} and *E*_*M*_ = ∪{(*S*_*i*_, *T*_*j*_) : *J*(*S*_*i*_, *T*_*j*_) ≥ *t*_*J*_, Δ_*C*_(*S*_*i*_, *T*_*j*_) ≥ *δ*}, whereby *J*(*S*_*i*_, *T*_*j*_) = |*S*_*i*_∩*T*_*j*_ | /|*S*_*i*_ ∪ *T*_*j*_| was the Jaccard similarity and Δ_*C*_(*S*_*i*_, *T*_*j*_) = |*d*_*c*_(*S*_*i*_) − *d*_*c*_(*T*_*i*_)| was the differential correlation density between *S*_*i*_ and *T*_*j*_, and *t*_*J*_ and *δ* were thresholds with 2/3 and 0.05 [15]. *J*(*S*_*i*_, *T*_*j*_) weighted every edge (*S*_*i*_, *T*_*j*_). We next identified the disrupted module pairs *ϒ*(*S*, *T*) by detecting the maximum weight matching in *M*, and we ranked them in nonincreasing order of their differential density Δ_*C*_. At last, we inferred genes involved in ccRCC as Γ = {*g*: *g* ∈ *S*_*i*_ ∪ *T*_*j*_, (*S*_*i*_, *T*_*j*_) ∈ *ϒ*(*S*, *T*)} and ranked in nonincreasing order of Δ_*C*_(*S*_*i*_, *T*_*j*_). To identify altered modules, we matched normal and ccRCC modules by setting high *t*_*J*_, which ensured that the module pairs either had the same gene composition or had lost or gained only a few genes.

### 2.4. Functional Enrichment Analysis

To further investigate the biological functional pathways of genes in altered modules from normal controls and ccRCC, Kyoto Encyclopedia of Genes and Genomes (KEGG) pathway enrichment analysis was performed by Database for Annotation, Visualization, and Integrated Discovery (DAVID) [25]. KEGG pathways with *P* value < 0.001 were selected based on EASE test implemented in DAVID. EASE analysis of the regulated genes indicated molecular functions and biological processes unique to each category [26]. The EASE score was used to detect the significant categories. In both of the functional and pathway enrichment analyses, the threshold of the minimum number of genes for the corresponding term > 2 was considered significant for a category

(5) $$\begin{array}{c} p=\frac{\left(\begin{array}{c} a+b\\ a \end{array}\right)\left(\begin{array}{c} c+d\\ c \end{array}\right)}{\left(\begin{array}{c} n\\ a+c \end{array}\right)}, \end{array}$$

where *n* was the number of background genes; *a*′ was the gene number of one gene set in the gene lists; *a*′ + *b* was the number of genes in the gene list including at least one gene set; *a*′ + *c* was the gene number of one gene list in the background genes; *a*′ was replaced with *a* = *a*′ − 1.

## 3. Results

### 3.1. Analyzing Disruptions in ccRCC PPI Network

**Figure 1:**
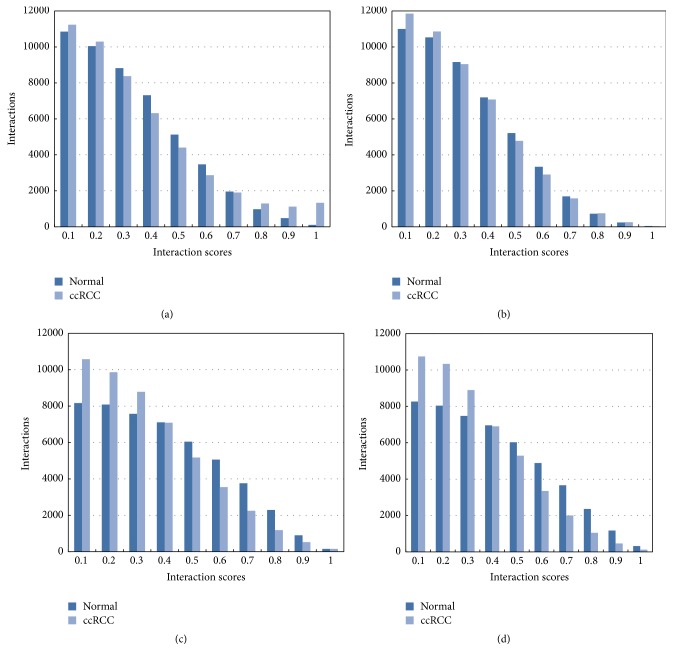
Score-wise distributions of interactions: normal versus ccRCC cases. (a) represents stage I of ccRCC, (b) represents stage II, (c) represents stage III, and (d) represents stage IV.

We obtained 20102 genes of normal and ccRCC cases after preprocessing and then investigated intersections between these genes' interactions and STRING PPI network and identified PPI networks of normal and ccRCC cases. The normal *H*_*N*_ and ccRCC *H*_*T*_ PPI networks of different stages (stages I, II, III, and IV) displayed equal numbers of nodes (8050) and interactions (49151). Although their interaction scores (weights) were different from each other, as shown in Figure 1, there was no statistical significance between normal and ccRCC cases in different stages in whole level based on Kolmogorov-Smirnov test (*P* > 0.05). However, the score distribution between the ccRCC networks and normal networks was different, especially for stages III and IV in the score distribution 0~0.3 (Figures 1(c) and 1(d)). Examining these interactions more carefully, distributions among different stages were also different, and changes of ccRCC networks and normal networks were more and more obvious from stage I to stage IV.

### 3.2. Analyzing Disruptions in ccRCC Modules

**Figure 2:**
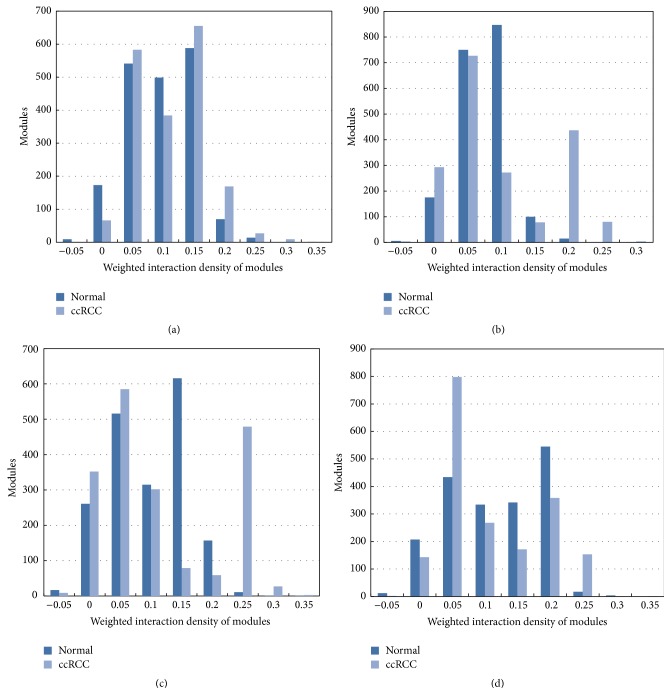
Weighted interaction density distribution of modules in normal and ccRCC cases. (a) represents stage I of ccRCC, (b) represents stage II, (c) represents stage III, and (d) represents stage IV.

Clique-merging algorithm was selected to identify disrupted or altered modules from normal and ccRCC PPI network in this paper. In detail, we performed a comparative analysis between normal *N* and ccRCC *T* modules to understand disruptions at the module level. Maximal cliques of normal and ccRCC PPI network were obtained based on fast depth-first algorithm, and maximal cliques with the threshold of nodes > 5 were selected for module analysis. Overall, we noticed that the total number of modules (1895), as well as average module sizes (20.235), was almost the same across the two conditions and four stages.  Table 1 showed overall changed rules of weighted interaction density between normal modules and ccRCC modules; we could find that maximal and average weighted density of normal case was smaller than that of ccRCC for each stage; in detail, the average weighted density of stages III (0.075) and IV (0.089) was a little higher than that of stages I (0.068) and II (0.046), while, in the overall level, the difference of module density scores had no statistical significance between normal and ccRCC cases in different stages with *P* > 0.05. Further, the relationship between modules weighted density distribution and numbers of modules was illustrated in Figure 2. The module numbers were different when the interaction density ranged from 0.05 to 0.25, especially for stages II, III, and IV of ccRCC. These differences might be the reasons of weighted density changes of ccRCC from different stages (Table 1).

**Figure 3:**
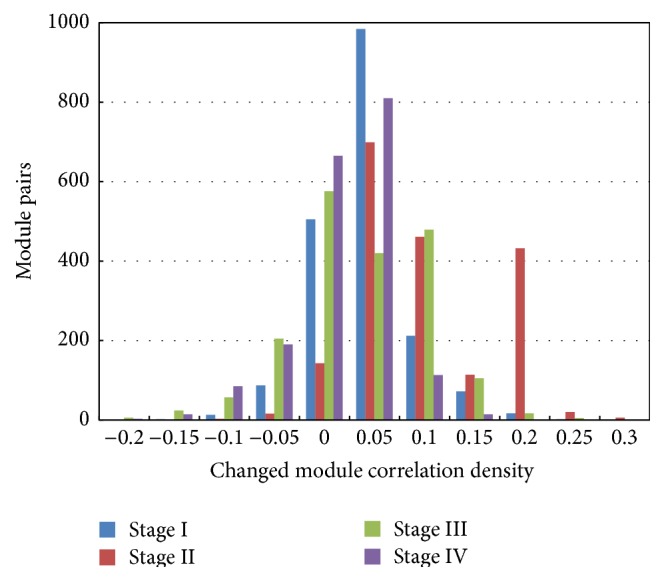
Module correlation density distributions of stage I, stage II, stage III, and stage IV.

Next, we obtained disrupted module pairs (ccRCC module and its relative normal module) based on modeling the set *ϒ*(*S*, *T*) as maximum weight bipartite matching and then calculated their PCC difference values (also called changed module correlation density value). With the thresholds *t*_*J*_ = 2/3 and *δ* = 0.05, the overall conditions of changed module correlation density of stages I, II, III, and IV in ccRCC had no significant difference (*P* > 0.05, Table 2). An overall decrease in maximum correlations of ccRCC modules with deepened stage was observed; besides minimum correlation density of stage III was the smallest among the four stages. In addition, changed module correlation density distributions were shown in Figure 3, and the number of modules was different in the same density interval of four stages, especially in the distribution interval of −0.05~0.20. For stage IV, module distributions firstly increased and then decreased with density increase; the maximum was reached at section of 0~0.05.

### 3.3. In-Depth Analyses of Disrupted Modules

When restricting random inspection correction of modules under condition of *P* < 0.01, we obtained 136, 576, 693, and 531 disrupted modules of stages I, II, III, and IV, respectively. Meanwhile, a total of 1026 genes were obtained of these disrupted modules, in detail, 317 genes of stage I, 450 genes of stage II, 658 genes of stage III, and 690 genes of stage IV. Therefore, 56 common genes existing in four stages were explored (Table 3), such as* MAPK1*,* CCNA2*, and* GSTM3*.

As we all know, differentially expressed (DE) gene was usually selected to screen significant genes between normal controls and disease patients; thus we identified 2781 DE genes between normal controls and ccRCC patients of four stages based on Linear Models for Microarray Data package. Taking the intersection of common genes and DE genes into consideration, we obtained 19 genes (*ALB*,* ASS1*,* GSTM3*,* MAD2L1*,* ALDH1B1*,* ALDH4A1*,* MAPK1*,* GSTZ1*,* GATM*,* FTCD*,* CCNA2*,* CENPF*,* GSTM4*,* ASNS*,* CCNB1*,* NAGS*,* ACY3*,* GSTA3*, and* ESPL1*), which might play important roles in the ccRCC development.

**Figure 4:**
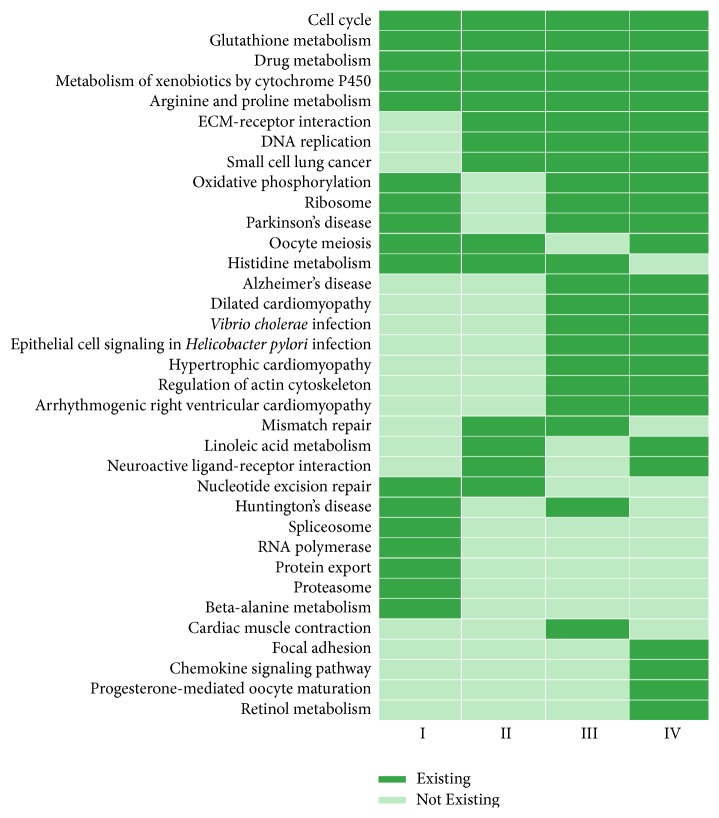
Distribution of pathways in stages I, II, III, and IV. Pathways were identified by KEGG with *P* < 0.001. The light green square represented the notion that one pathway did not exist in the stage, while the dark one stood for the notion that the pathway existed in the stage.

Pathway enrichment analysis based on genes in disrupted modules of different stages was performed, and the results within threshold *P* value < 0.001 were shown in Figure 4; there were 5 common pathways (glutathione metabolism, cell cycle, alanine, aspartate, and glutamate metabolism, arginine and proline metabolism, and metabolism of xenobiotics by cytochrome P450) across stages I, II, III, and IV.

## 4. Discussions

The objective of this paper is to identify dysregulated genes and pathways in ccRCC from stage I to stage IV according to systematically tracking the dysregulated modules of reweighted PPI networks. We obtained reweighted normal and ccRCC PPI network based on PCC and then identified modules in the PPI network. By comparing normal and ccRCC modules in each stage, we obtained 136, 576, 693, and 531 disrupted modules of stages I, II, III, and IV, respectively. Furthermore, a total of 56 common genes (such as* MAPK1* and* CCNA2*) and 5 common pathways (e.g., cell cycle, glutathione metabolism, and arginine and proline metabolism) of the four stages were explored based on gene composition and pathway enrichment analyses. The common genes and pathways from stage I to stage IV were significant for ccRCC development; if we control these signatures and the biological progress in the early stage of the tumor, there might be positive effects on the therapy.

*MAPK1* (mitogen-activated protein kinase 1), which encoded a member of the* MAPK* family, acted as an integration point for multiple biochemical signals and was involved in a wide variety of cellular processes such as proliferation, differentiation, transcription regulation, and development [27]. Roberts and Der had reported that aberrant regulation of* MAPK* contributed to cancer and other human diseases, such as ccRCC; in particular, the* MAPK* had been the subject of intense research scrutiny leading to the development of pharmacologic inhibitors for the treatment of cancer [28]. Moreover,* MAPK* participant biological processes were key signaling pathways involved in the regulation of normal cell proliferation and differentiation. For example, an increase in the activation of* MAPK* signal transduction pathway was observed as the cancer progresses [29].* MAPK*/extracellular signal-related kinase pathway was activated in tumors and represented a potential target for therapy [30]. Therefore, ccRCC as a common tumor was related to* MAPK* closely.

**Figure 5:**
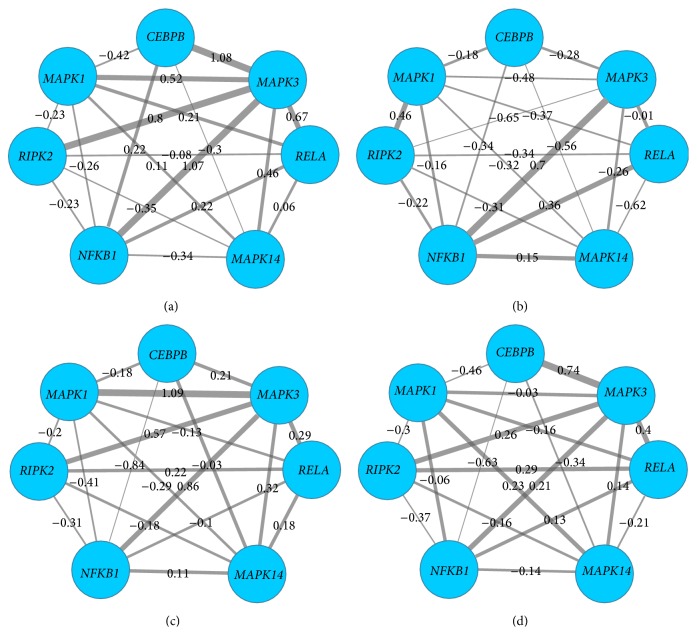
Swapping behavior in altered module (*MAPK1*,* CEBPB*,* MAPK3*,* RELA*,* MAPK14*,* NFKB1*, and* RIPK2*). Nodes stood for genes, and edges stood for the interactions of genes. The thickness of the edges represented the interaction scores or expression levels between two genes in the module, more thickness with higher value of expression scores. (a) represents stage I of ccRCC, (b) represents stage II, (c) represents stage III, and (d) represents stage IV.

**Figure 6:**
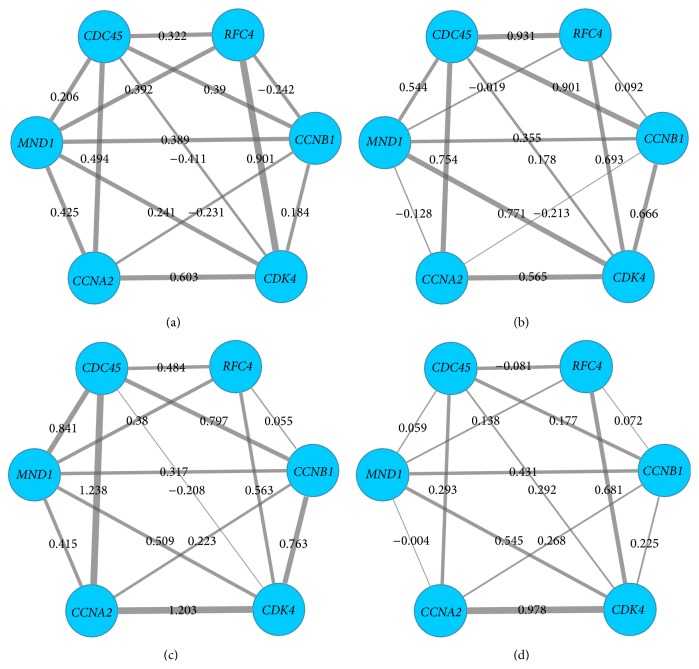
Swapping behavior in altered module (*CCNA2*,* CDK4*,* CDC45*,* RFC4*,* CCNB1*, and* MND1*). Nodes stood for genes, and edges stood for the interactions of genes. The thickness of the edges represented the interaction scores or expression levels between two genes in the module, more thickness with higher value of expression scores. (a) represents stage I of ccRCC, (b) represents stage II, (c) represents stage III, and (d) represents stage IV.

Furthermore, we studied gene swapping behaviors in single altered module of four stages, taking* MAPK* family genes related altered modules as an example. As shown in Figure 5, we could discover that, for a module (*MAPK1*,* CEBPB*,* MAPK3*,* RELA*,* MAPK14*,* NFKB1*, and* RIPK2*) in stages I, II, III, and IV, its gene compositions (nodes) were the same, but the interaction scores (edges) were different. The interaction value between* MAPK1* and* MAPK3* was 0.52, −0.48, 1.09, and −0.03 in stages I, II, III, and IV, respectively, and there was a weak correlation of the two genes in stage II. It might explain differences of modules and existence of dysregulated modules. Swapping behavior in the altered module (*CCNA2*,* MND1*,* CDC45*,* RFC4*,* CCNB1*, and* CDK4*) was shown in Figure 6.

*CCNA2*, cyclin A2, was expressed in dividing somatic cells and regulated cell cycle progression by interacting with cyclin-dependent kinase (*CDK*) kinases [31]. Consistent with its role as a key cell cycle regulator, expression of* CCNA2* was found to be elevated in a variety of tumors such as breast, cervical, liver, and kidney tumors [32]. It was not clear whether increased expression of* CCNA2* was a cause or result of tumorigenesis;* CCNA2*-*CDK* contributed to tumorigenesis by the phosphorylation of oncoproteins or tumor suppressors [33]. In our paper, we had also proved that the correlation value between* CCNA2* and* CDK4* of the four stages was 0.603, 0.565, 1.203, and 0.978 in sequence (Figure 6). We might infer that cell cycle played a medium role in correlations of* CCNA2* and cancers; thus cell cycle was discussed next.

Cell cycle is the series of events that take place in a cell leading to its division and duplication, and dysregulation of the cell cycle components may lead to tumor formation [34]. It had been reported that alterations in activated proteins (cyclins and cyclin-dependent kinases, etc.), which led to failure of cell cycle arrest, may thus serve as markers of a more malignant phenotype and cell cycle-related genes aided in discrimination of atypical adenomatous hyperplasia from early adenocarcinoma [35]. Chen et al. demonstrated that cell cycle progression effects on* NF-κB* activity represented a molecular basis underlying the aggressive tumor behavior [36]. Besides, cell cycle checkpoint inactivation allowed DNA replication in aneuploid cells and may favor oncogenic genomic [37], and a cell cycle regulator is potentially involved in genesis of many tumor types, such as ccRCC [38]. We could conclude that cell cycle played a key role in the ccRCC progress.

Our results also showed that ccRCC had close relationship with metabolism pathways, such as glutathione metabolism and arginine and proline metabolism. Glutathione metabolism which played both protective and pathogenic roles in cancers was crucial in the removal and detoxification of carcinogens [39]. And the present review highlighted the role of glutathione and related cytoprotective effects in the susceptibility to carcinogenesis and in the sensitivity of tumors to the cytotoxic effects of anticancer agents [40]. Recently, Hao et al. discovered that three significant pathways related to ccRCC, namely, arginine and proline metabolism, aldosterone-regulated sodium reabsorption, and oxidative phosphorylation, were observed [41]. Arginine/proline metabolism is a significant pathway in ccRCC that had been discovered by Perroud et al. previously [42], and the results were in accordance with our analysis.

## 5. Conclusions

In conclusion, we successfully identified dysregulated genes (such as* MAPK1* and* CCNA2*) and pathways (such as cell cycle, glutathione metabolism, and arginine and proline metabolism) of ccRCC in different stages, and these genes and pathways might be potential biological markers and processes for treatment and etiology mechanism in ccRCC.

## Figures and Tables

**Table 1:** Correlations of normal and ccRCC modules of different stages.

Module set	Stage I		Stage II		Stage III		Stage IV	
	Normal	ccRCC	Normal	ccRCC	Normal	ccRCC	Normal	ccRCC
PCC correlation								
Maximal	0.315	0.324	0.254	0.278	0.324	0.339	0.294	0.326
Average	0.068	0.083	0.046	0.074	0.075	0.087	0.089	0.084
Minimum	−0.076	−0.072	−0.073	−0.073	−0.092	−0.074	−0.078	−0.057

**Table 2:** Overall conditions of changed module correlation density of ccRCC stages.

ccRCC stages	Changed module correlation density		
	Maximum	Average	Minimum
I	0.255	0.015	−0.195
II	0.254	0.028	−0.192
III	0.253	0.012	−0.246
IV	0.155	−0.006	−0.240

**Table 3:** Common genes of disrupted modules based on four ccRCC stages.

Number	Genes
1	*MAPK1*
2	*CDC6*
3	*CDKN1A *
4	*SF3B6*
5	*CPSF3*
6	*SRSF6*
7	*SRSF1*
8	*U2AF1*
9	*SRSF4*
10	*CCNB1*
11	*ESPL1*
12	*NCAPH*
13	*KIF11*
14	*BUB1B*
15	*CDC20*
16	*CCNA2*
17	*CCNB2*
18	*MAD2L1*
19	*CENPF*
20	*ALDH4A1*
21	*NCBP1*
22	*MGST1*
23	*GSTZ1*
24	*GSTM2*
25	*GSTM5*
26	*GSTM3*
27	*GSTA3*
28	*SNRPD3*
29	*CDKN1B*
30	*NDUFAB1*
31	*RNPS1*
32	*ALB*
33	*LPA*
34	*GOT1*
35	*GLUD1*
36	*FTCD*
37	*GLUD2*
38	*ALDH3A2*
39	*ALDH9A1*
40	*ALDH1B1*
41	*ALDH7A1*
42	*NAGS*
43	*GATM*
44	*ASNS*
45	*ACY3*
46	*ASPA*
47	*GOT2*
48	*ASS1*
49	*GAD2*
50	*ALDH2*
51	*MGST2*
52	*MGST3*
53	*GSTO1*
54	*GSTM4*
55	*RPA3*
56	*UPF3B*
